# Developmental programming: the role of growth hormone

**DOI:** 10.1186/s40104-015-0001-8

**Published:** 2015-02-12

**Authors:** Anita M Oberbauer

**Affiliations:** Department of Animal Science, University of California, One Shields Ave, Davis, CA 95616 USA

**Keywords:** Epigenetic, Growth hormone, Programming, Transgeneration

## Abstract

Developmental programming of the fetus has consequences for physiologic responses in the offspring as an adult and, more recently, is implicated in the expression of altered phenotypes of future generations. Some phenotypes, such as fertility, bone strength, and adiposity are highly relevant to food animal production and *in utero* factors that impinge on those traits are vital to understand. A key systemic regulatory hormone is growth hormone (GH), which has a developmental role in virtually all tissues and organs. This review catalogs the impact of GH on tissue programming and how perturbations early in development influence GH function.

## Introduction

Broadly considered, development has two distinct yet interrelated facets: alterations in gene expression associated with the normal developmental profile of an organism and alterations associated with developmental plasticity permitting adaptation to environment perturbations. The fundamental role of normal fetal development is best exemplified by pattern formation in the embryo. “Programming” was a term coined in 1986 to reflect that events occurring *in utero* alter adult phenotypic and metabolic traits [1]. The influence of fetal and neonatal nutritional environments on metabolic programming was first conceptualized by Lucas [2] and the concept of developmental programming was later expanded to include other environmental perturbations [3,4]. The impact of environmental challenges experienced in the neonatal period emphasizes the need to understand both inherent development as well as the mechanistic processes by which developmental programming is achieved.

Recent data highlight that the metabolic environment experienced by the fetus influences phenotypic expression and disease susceptibility in later life [5,6]. Small for gestational age (SGA) offspring, often arising as a consequence of malnourished mothers, is correlated with adult hypertension, glucose intolerance, insulin resistance, type 2 diabetes, dyslipidemia, and diminished measures of bone strength. Metabolic bone disease is also associated with prenatal nutrient deprivation [7]. Adult-onset disorders associated with SGA neonates represent programming occurring at the gene level by methylation, gene silencing, and other epigenetic modifications established during fetal development (reviewed in [8]). It is worth noting that the definition of epigenetics used in this review is “the structural adaptation of chromosomal regions so as to register, signal or perpetuate altered activity states” first proposed by Bird in 2007 to encompass the many aspects of epigenetic alterations [9].

Although not developmental programming, the most familiar illustration of developmental epigenetic imprinting is the insulin-like growth factor-2 (IGF-2) pathway. The methylation status differs between the paternally and maternally inherited *Igf-2* gene with the paternal copy as the sole source of IGF-2 during development and the maternally inherited copy silenced. It is hypothesized that the maternal and paternal expression are balanced to maximally promote fetal growth while preventing excessive depletion of the dam’s resources [10]. Another example of the role of epigenetic imprinting occurs in Prader-Willi Syndrome, a syndrome characterized by growth hormone (GH) insufficiency [11]. The genetic cause is the deletions of paternal chromosome 15; the maternal region is silenced by epigenetic factors. While IGF-2’s role is predominantly in prenatal development, the key postnatal hormone is GH. Growth hormone effects are either direct or mediated through the induction of IGF-1 [12] to regulate overall body growth through its effects on adipose, bone, and muscle, is growth hormone (GH) [13].

Importantly, developmental programming may have multigenerational consequences transmitted through epigenetic modifications of the genome. Transgenerational epigenetic programming was initially recognized as a result of malnourishment. The Dutch famine, sometimes referred to as the “Hunger winter”, is most often cited as the initial epidemiological human case study illustrating the impacts of nutritional stress upon both physical and behavioral traits in subsequent generations (reviewed in [14]). More recent evidence of transgenerational consequences has been associated with environmental perturbations including toxicants and hormonal manipulations [15-17]. Although many effects are termed “transgenerational,” Heard and Martienssen [18] recommend a more parsimonious use of the term and define much of what has been observed as “intergenerational” which will be employed in this review. Nevertheless, understanding the link between fetal and neonatal programming and subsequent phenotypic expression is important for human health and has value for sustainable food animal production systems. Nutritional, physical, and psychological stress experienced by livestock influences immediate productivity and health [19-21] and yet notably can also affect future generations’ production through developmental programming.

## Review

### Role of GH in normal development

The accepted role of GH and the GH-IGF axis in tissue development is predominantly postnatal with other hormones assuming importance *in utero* [5]. Yet GH is known to influence fetal development. For example, transgenic mice overexpressing GH, produce offspring with a 12% reduction in birth weight [22] and calves born to dams given exogenous bovine somatotropin (GH) have reduced birth weights [23,24]. These findings indicate that GH, or its downstream regulators, plays a significant role in normal fetal development. The intimate link between GH and generalized growth has led to the speculation that GH may also affect the expression of IGF-2 though the evidence does not support this supposition to any great extent [25].

Evidence that fetal perturbations program GH signaling is also emerging. A common model used to define the role of GH in developmental programming is the neonate that experienced impaired fetal growth, specifically SGA infants, who are characterized by compromised bone growth and reduced body mass. Infants having low birth weights trend to lower circulating GH as young adults [26] indicating persistent effects by early developmental experiences on GH secretion. Despite prenatal growth impairment, approximately 90% of SGA births have accelerated growth in the first two postnatal years achieving heights in the normal range [27,28]; however the remaining 10% do not exhibit catch-up growth giving evidence of fetal programming of the GH-IGF endocrine axis as a consequence of early growth dysfunction [29]. Also associated with SGA is reduced bone mass and strength in adulthood [30] indicative of developmental programming of bone remodeling. GH, with its role in bone mineral density [31], may mediate the adult bone characteristics arising from *in utero* growth restriction. Furthermore, adult height is correlated with placental GH expression. Genetic variation within the sequence of the placental-form of GH is significantly associated with mature height confirming that altered expression of GH *in utero* governs longitudinal bone growth potential [32]. Immune function is depressed chronically in SGA rat pups, although the condition can be reversed by provision of exogenous GH during the pre-weaning growth stage [33] indicating GH is important in the ontogeny of immune competence.

Birth weight and neonatal growth is predictive of adult circulating GH levels suggesting that the intrauterine environment programs GH secretion [34] or tissue responsiveness to GH. Waldman and Chia postulate that idiopathic short stature reflects epigenetic changes to the GH receptor or to IGF-1 genes [35]. Research exploring the chromatin landscape suggests a tissue specificity in the accessiblity of the IGF-1 promoter indicating epigenetic regulation of the GH-IGF-1 axis [36,37]. This view is supported by developmental profiling of IGF-1 mRNA expression in cattle; as female calves mature, IGF-1 gene expression in the pituitary is reduced, particularly the exon most responsive to GH, whereas IGF-1 expression in the uterus increased [38].

### Elevated GH models

#### In utero exposure

The role of GH in normal development was established decades ago through ablation studies and assessing the physiological consequences of the absence of GH. Additional models to characterize GH action elevate the hormone either through exogenous administration of GH, GH-transgene expression, or chemical compounds that induce GH. Elevated GH *in utero* reduces birth weights of calves [23], lambs [39], and rodents [22]. In sheep, fetal adipogenesis is enhanced when dams are exposed to GH indicating the direct effect of GH on adipose metabolism [39]. For rodent models, neonates are both lighter in weight and have reduced skeletal size at birth. In contrast, pregnant sows given hydroxyl beta methylbutyrate (HMB), a compound that enhances fetal GH and IGF-1, give birth to piglets with increased birth weights [40]. Furthermore, the piglet data indicate that fetal exposure to elevated GH has persistent effects on growth parameters into adulthood; they had increased growth rate to slaughter, lean carcass content and indices of bone strength [40]. Similar to the rodents, piglets from HMB treated sows had shorter femurs although in a different study, provision of GH directly to pregnant sows did not affect bone length in the neonates [41]. Ewes treated with extended -release GH during gestation delivered lambs having heavier birth weights and a sustained postnatal growth advantage over lambs born of untreated ewes as well as a blunted response to stimulation of the GH axis [42,43]. Taken together, the evidence suggests that exposure to elevated GH *in utero* exerts long term consequences on the offspring possibly through alteration of maternal metabolic pathways and placental function.

#### Postnatal exposure

Elevated GH postnatally has significant effects on bone, muscle, and adipose. Human SGA infants given GH respond with increased bone growth velocity for the duration of GH treatment [8]. In rodents, provision of GH during the postnatal period can reverse the *in utero* growth inhibition and restore overall bone length [22]. GH exerts stimulatory effects on linear growth rates in rodents with transient elevation of GH increasing bone growth rate. At the cellular level, GH accelerates bone growth by hyperplasia with little impact on hypertrophy [44]. Provision of elevated GH in a GH-transgenic mouse model also increases the duration of bone elongation though the degree of responsiveness is sensitive to the chronological age of exposure with early neonatal tissue more responsive than slightly older ages.

Lambs given HMB during the first 3 postnatal weeks show elevated circulating levels of GH, IGF-1, and biochemical markers of bone turnover. However once the HMB treatment concludes, these indices all fall to control values indicating resistance to long term bone programming by HMB when provided postnatally [45]. The failure of persistent HMB treatment effects in lambs, in contrast to the direct GH effects seen in rodents and pigs, may reflect the precocial development of sheep when compared to that of rodents and pigs suggesting that the period responsive to GH programming may be *in utero* for lambs.

The enhanced gain accompanying elevated GH has a greater proportion of protein than normal tissue accrual in rodents. Rodent development is characterized by each unit of gain being composed of the same proportions of water, lipid, protein and ash across the entire growth phase, despite the speed of accrual [46]. In contrast, under conditions of elevated GH, protein comprises a larger proportion of gain [47]. This preferential repartitioning by GH of nutrients to muscle is also seen in pigs transgenic for GH [48], broilers supplemented with HMB [49], fish given exogenous GH [50], and GH-transgenic fish [51]. Exogenous GH increases protein anabolism by enhancing protein synthesis [52,53].

Therapeutic GH treatment of SGA children offers growth advantages as noted above, yet the elevated GH also reduces insulin sensitivity potentially increasing the risk of diabetes. Recent studies of children born SGA and treated with GH would suggest this is not a valid concern as they have normal insulin post-GH treatment [8]. However, these children have not aged to a point wherein the long term consequences of GH treatment can be evaluated; thus, adult onset diabetes remains a concern. In a rodent model, elevating GH in adult mice increases circulating insulin with levels remaining elevated even after reduction of GH consistent with an insulin resistant state, though persistence of this condition was not determined [54].

The role of GH in adipogenesis is complex. *In utero* or postnatal exposure to elevated GH in a GH-transgenic mouse model increases adipose storage by increasing adipocyte hyperplasia, differentiation, and cellular lipid content [39,54,55]. Artificially elevating GH induces lipoprotein lipase which in turn stimulates adipogenesis; the elevated GH also increases circulating insulin which then can induce lipoprotein lipase further promoting adipose storage [56]. Postnatal exposure to GH acts on membrane lipids to program response to cellular perturbations. Elevated GH in rodents creates a more unsaturated lipid profile in cell membranes by activating membrane desaturase pathways [57]. This net flux through the lipid metabolism pathways generates eicosanoids important in the inflammation process [58].

The interplay with leptin adds more complexity to the influence of GH on adipose. Leptin, synthesized by adipose cells, regulates energy metabolism (reviewed in [59]) and influences fetal brain and bone development [60]. Leptin also defines energy storage adequacy during neonatal life and can program the neuronal regulation of adult food intake and satiety with leptin programming in early development altering adiposity at later ages [61,62]. Newborn rodents and piglets born SGA experience adipogenesis however neonatal provision of exogenous leptin can reverse the adipocyte proliferation induced by intrauterine growth restriction [61,63]. Leptin levels correlate with birth weight with SGA infants having low leptin levels while large for gestational age babies have high leptin concentrations [64]. Although the correlation is largely independent of the GH-IGF-1 axis (reviewed by [62]), GH levels influence maternal and fetal leptin levels. In sheep, fetal adipogenesis is enhanced when dams are exposed to GH and leptin levels are reduced in the dam and the fetus, demonstrating a direct effect of GH on adipose *in utero* [39] with potential of long term programming impacts.

Early dysregulation of GH promotes adipogenesis which in turn elevates leptin that influences adipose function at later ages. Elevated GH in a GH-transgenic mouse model increases plasma leptin [65] while mice transiently exposed to elevated GH during early postnatal development become obese having increased circulating leptin once the excess GH is withdrawn [54]. In these GH-transgenic mice, although each adipocyte expresses less leptin, the leptin in circulation is greater due to the increased overall adipocity. This suggests that prolonged elevation of GH disrupts normal physiological responses to elevated plasma leptin, creating leptin resistance and obesity; in the case of elevated GH, the most likely site of leptin signaling impairment is at the hypothalamic axis. Supporting this view of GH generating leptin resistance is that the elevated GH in the GH- transgenic mouse increases plasma leptin as well as the leptin receptor (NPY) gene and protein expression [65]. It is proposed that leptin resistance can be programmed during fetal and neonatal life with long term impacts on body energy stores potentiating adult onset metabolic disorders [63].

### Models of *in utero* programming

Studies in multiple mammalian species demonstrate that aberrant programming of adult tissue response is associated with stress-induced glucocorticoid secretion during fetal and perinatal development. Maternal stress in humans is known to impact neurological circuitry of the offspring: infants have an increased risk of neuropsychiatric disease when the mother experienced psychological stress during the first trimester (reviewed in [66]). Similarly, prospective stress research with rodents and non-human primates have identified gestational periods of increased susceptibility to developmental disruption [66]. Importantly the maternal stress response is characterized by altered methylation patterns in the fetal DNA [67] thereby programming future gene expression patterns that may also be transmitted to the next generation. Similar changes in methylation patterns have been detected in response to maternal exposure to toxins and hormones [68] resulting in intergenerational epigenetic changes having significant impact on future physiological performance and significant implications on selection for production species.

Early research demonstrated that adult GH secretory patterns are influenced by perturbations during the perinatal period. For example, male rats normally express a high amplitude secretory pattern of GH whereas females have low amplitude pulses within a higher basal background. Transient manipulation of sex steroids in the neonatal period can modify the GH secretory pattern to that of the opposite sex [69]. Glucocorticoids are viewed as critical modulators of GH in a wide range of species including chickens, rodents, and humans with impacts on the hypothalamus and pituitary regulation of GH release, somatotrope development, and peripheral tissue responsivity [70-74]. In turn, GH mediates gene regulation by increasing accessibility of the transcriptional machinery by chromatin remodeling of promoters through histone acetylation [36]. Taken together, stress has profound impacts on the development of the neonate that may transcend a single generation and GH plays a role in programming the consequences of the stress.

#### Nutritional stress

Mothers who are undernourished during pregnancy give birth to SGA infants and when adult those children exhibit adult-onset obesity, insulin resistance, hypertension, and metabolic dysfunction. Maternal nutritional deprivation stress also is implicated in intergenerational epigenetic programming to alter future generations’ growth and metabolic phenotypes. This phenomenon is suggested to account for the rising cases of human obesity, diabetes, and coronary disease (reviewed in [66]).

Levels of placental GH found in maternal circulation are positively correlated with fetal birth weight and in times of maternal nutrient deprivation and SGA pregnancies, placental GH secretion is reduced [32]. A recent study of ewes undernourished during gestation reported that although maternal circulating GH and placental weight were not reduced [75], birth weights and crown rump lengths were [76]. Newbern and Freemark [77] review the evidence that supports a direct role of GH and placental GH in the programming of fetal growth and long-term metabolic function. Some evidence is based upon transgenic mice that overexpress placental GH and their high birth weight pups with enhanced growth capacity relative to their non-transgenic littermates [78].

As noted above, there are generalized consequences of SGA on bone characteristics but SGA due to nutritional deprivation also programs bone through the GH- IGF axis. The necessity of adequate fetal and neonatal nutrition for normal bone growth and adult bone integrity was underscored by Eastell and Lambert in their review [79]. In rats, post-weaning dietary protein restriction alters the GH secretory profile, blunting the peak GH pulses [80] which alters growth and metabolic responses. It is known that neonatal nutrition programs adult bone mass [81] and bone density is positively associated with peak GH and IGF-1 concentrations but elevated median GH concentrations, as seen with dietary restriction, is negatively associated with bone density [34]. Presumably the effects on bone are due to the role that GH plays in opening the chromatin surrounding the IGF-1 gene. Moreover, maternal malnutrition alters methylation of the GH responsive promoter 2 of the IGF-1 gene (reviewed in [12]) and intrauterine growth restriction is associated with sustained hypermethlyation of the IGF-1 promoter in rats [82] thereby amplifying the signaling impairment of GH in the neonate.

Another mechanism of nutritional programming on bone is by its effects on the formation of the embryonic skeletal anlage. Fetal and postnatal bone growth depends upon chondrocyte hypertrophy and hyperplasia. Fibroblast growth factors (FGF) and their cognate fibroblast growth factor receptors (FGFR) are key signaling molecules of chondrocyte function in developing bone. Activation of different FGFR family members both promote and inhibit chondrocyte proliferation [83]. The FGF21 ligand directly inhibits chondrocyte proliferation and antagonizes the proliferative effects of GH on growth plate chondrocytes [84] thereby impairing chondrogenesis and bone growth. FGF21 is increased during food restriction and attenuates the action of GH at the chondrocytic level and at the systemic level by impairing IGF-1 expression [84]. Antagonizing GH action by elevated FGF as result of food restriction has implications for many of the body systems because of GH’s extensive broad range of target tissues.

In rats, restricting maternal dietary protein to induce intrauterine growth restriction enlarges abdominal adipose depots when animals are adult [85]. The enlarged adipose depots upregulate expression of genes associated with enhanced lipid metabolism and adipose accrual. Provision of exogenous GH postnatally can minimize those adult conditions [33,86]. Specifically, adult-onset obesity seen in pups born to undernourished rat dams can be prevented with preweaning GH treatment [87] (reviewed in [88]). This provides compelling evidence of early GH programming the adult physiological response.

Intrauterine growth restricted babies, piglets, and rodent pups have reduced leptin at birth and then propensity for obesity at adulthood [89]; provision of leptin reverses this tendency [61]. At birth infants considered SGA have greater methylation of the leptin promoter correlating with the reduced circulating leptin levels [90]. In contrast, using a mouse model, early nutritional programming of the fetus by low protein diets is correlated with hypomethylation of the leptin gene that is maintained throughout life [91]. In the latter study, the authors acknowledge their results differ from the pattern of leptin expression, and methylation, observed in other species in response to maternal malnutrition. Adipocyte hypertrophy results in hypomethylation of the leptin gene and Increases leptin expression which increases adipose hyperplasia [89]. Clearly the specifics of the nutritional deprivation, timing, and species evaluated have significant impact on the manifestation of maternal malnutrition on adult susceptibility to metabolic disruption.

#### Physical stress

Physical stressors, often modeled with induced hypoxia, have been used to assess the epigenetic consequences of stress. Hypoxia-inducible factor 1 (HIF-1), induced under conditions of hypoxia to adapt to reduced oxygen supply, is also required for normal fetal tissue and skeletal development [92]. The transcription factor HIF-I coordinates with additional factors to induce chromatin remodeling of target genes [93]. In addition to the role of HIF-1 in hypoxia adaptation, in rodents the GH axis modulates the hypoxic stress response [94]. In periods of intermittent hypoxia, GH is neuroprotective [95]. However, hypoxia elevates glucocorticoid expression from the fetal adrenal gland [96] and elevated glucocorticoids can lead to the programming of the fetal GH axis as detailed earlier in this article [73,74]. Furthermore, hypoxia inhibits GH release by enhancing the expression of somatostatin, the hypothalamic GH inhibitor [97]. Varvarigou et al [98] propose that severe hypoxia permanently damages the fetal hypothalamic-hypophyseal axis leading to greatly diminished GH secretory profiles. Despite the knowledge that fetal programming is a consequence of hypoxia, defining the long term and intergenerational effects of physical stressors are yet to be fully explored.

## Conclusions

Growth hormone has a pivotal role in pre and postnatal development although its effects on intergenerational programming are as yet under studied. Given the plasticity of postnatal development, defining fetal and neonatal programming and the effect upon later phenotypes is important to maximize the expression of desirable traits in food animals. This is particularly important on the impact of more permanent epigenetic alterations of gene expression that may have future consequences. Historically, classical genetic selection has permitted extremely favorable phenotypic advances. The implementation of quantitative trait loci selection schemes for livestock based upon genomic signatures will accelerate genetic improvement. Overlaid upon genetic selection one must be mindful of the epigenetic programming that environmental perturbations can exert on future trait expression and how that may factor into selection schemes for agricultural livestock production in a changing environment. Better defined knowledge of the mechanisms of programming will facilitate incorporation of epigenetic factors into selection.

## Abbreviations

GH: Growth hormone
HIf-1: Hypoxia-inducible factor 1
HMB: Hydroxyl beta methylbutyrate
IGF-1: Insulin-like growth factor 1
FGF: Fibroblast growth factors
FGFR: Fibroblast growth factor receptors
SGA: Small for gestational age
